# Feasibility of a semiautomated, individualized coaching intervention for glycemic management in adults with type 1 diabetes: a pilot randomized controlled trial

**DOI:** 10.1007/s40618-026-02835-1

**Published:** 2026-03-07

**Authors:** Seohyun Kim, Minkyeong Kim, Sang Ho Park, Soojin Park, You-Bin Lee, Sang-Man Jin, Kyu Yeon Hur, Jae Hyeon Kim, Gyuri Kim

**Affiliations:** 1 Division of Endocrinology and Metabolism, Department of Medicine, Sungkyunkwan University School of Medicine, Samsung Medical Center, Seoul, Republic of Korea; 2 Department of Clinical Research Design and Evaluation, Samsung Advanced Institute for Health Sciences and Technology, Sungkyunkwan University, Seoul, 06355 Republic of Korea

**Keywords:** Type 1 diabetes, Coaching, Algorithms, Smartphone

## Abstract

**Background:**

In the Korean Type 1 Diabetes Home Care Pilot Project, patients with type 1 diabetes (T1D) receive at least two monthly remote educational consultations from a multidisciplinary team. This randomized trial evaluated the feasibility and acceptability of individualized, semi-automated coaching messages based on continuous glucose monitoring (CGM) data for glycemic and emotional management in patients with T1D as a potential alternative to current treatments.

**Methods:**

Participants who had already enrolled in the national home care program for T1D were randomized in a 1:2 ratio to the control or intervention groups. The control group continued their current treatment, while the intervention group received weekly CGM-based coaching messages for glycemic management and biweekly emotional support messages based on the Patient Health Questionnaire-9 (PHQ-9) for six weeks. Feasibility was assessed based on the difference in time in range (TIR; 70–180 mg/dL) at week 6, other CGM metrics, and patient-reported outcomes.

**Results:**

Eighteen participants were enrolled. At week 6, the median TIR was 72.6% (interquartile range [IQR], 46.8–77.0) in the control group (*n* = 6) and 79.0% (IQR, 65.0–82.1) in the intervention group (*n* = 12), with no significant difference (*p* = 0.33). The intervention group showed a significantly lower median PHQ-9 score (2.5) in week 6 compared to the control group (6.0) (*p* = 0.03).

**Conclusion:**

The use of individualized semi-automated coaching messages was feasible and acceptable for glycemic and emotional management in individuals with T1D. Further large-scale studies are needed to confirm these findings and assess their long-term clinical impact.

**Trial registration:**

ClinicalTrials.gov (NCT06525454).

**Supplementary Information:**

The online version contains supplementary material available at 10.1007/s40618-026-02835-1.

## Introduction

Type 1 diabetes (T1D) is an autoimmune disease characterized by absolute insulin deficiency, requiring lifelong insulin therapy and careful glycemic management to prevent hypo- and hyperglycemia [1]. Due to the challenges in insulin titration and glycemic control, individuals with T1D face more than twice the risk of all-cause mortality and myocardial infarction, and three times the risk of end-stage renal disease compared to individuals with type 2 diabetes [2, 3].

Recent American Diabetes Association guidelines recommend continuous glucose monitoring (CGM) as the method for monitoring blood glucose levels in patients with T1D [4]. In real-world settings, CGM use has been associated with a 0.1% reduction in glycated hemoglobin every 3 months in individuals with T1D, compared to self-monitoring of blood glucose (SMBG) [5]. Despite the efficacy of CGM, its use presents challenges, including discomfort, cost, and the time required for its application, as well as the difficulty in interpreting CGM data [6]. Applying CGM data to insulin titration can be challenging for patients. A recent study found that providing personalized education on CGM use in patients with T1D was associated with a significant reduction in glycated hemoglobin [7].

Adults with T1D are approximately three times more likely to experience depression compared to individuals without diabetes. People with T1D who also have psychiatric disorders have a 3%–40% increased risk of cardiovascular disease or death compared to individuals without diabetes [8, 9]. A recent national cohort study indicated that the probability of suicide among individuals with T1D may exceed that of individuals with cancer, underscoring the need for interventions to mitigate the psychological burden of T1D [10]. A previous study reported that over a 9-month period, 54.4% of individuals with T1D experienced elevated diabetes distress, indicating the need for ongoing monitoring of psychological burden [11].

In the Republic of Korea, the Type 1 Diabetes Home Care Pilot Project, launched in 2020 by the Ministry of Health and Welfare, supports individuals with T1D through a multidisciplinary approach. Through a team of diabetes care professionals including physicians, nurses, and nutritionists the program provides in-person education and at least two monthly remote educational consultations via phone calls or interactive messaging to improve glycemic control and prevent diabetes-related complications. A recent retrospective cohort study demonstrated the program’s effectiveness in improving glycemic control, with participants achieving greater reductions in HbA1c levels than those receiving usual care [12]. However, the program’s reliance on intensive human involvement limits its scalability and long-term sustainability, as reviewing CGM data and providing individualized feedback require significant time and clinical resources. Therefore, we developed a novel semiautomated digital intervention designed to support glycemic control and psychological well-being through data-driven coaching. In this randomized feasibility trial, we aimed to evaluate the acceptability and practicality of delivering weekly CGM-based text and plot summaries along with algorithm-generated coaching messages tailored to insulin management over a 6-week period. Additionally, to address the often-overlooked emotional burden of T1D, the intervention included a 2-week depressive symptom assessment, followed by individualized visual summaries and supportive messages. This study thus examined whether a system delivering personalized, algorithm-driven coaching messages derived from CGM data could serve as a feasible and acceptable strategy to support glycemic control and emotional well-being in individuals with T1D, as a potential alternative to the national home care pilot program. Therefore, this feasibility study was designed primarily to evaluate the feasibility and acceptability of delivering a semiautomated, CGM-based coaching intervention, rather than to assess its clinical effectiveness.

## Research design and methods

### Study design

This single-center, randomized, feasibility, and acceptability trial was conducted at the Diabetes Center, Division of Endocrinology and Metabolism, Samsung Medical Center (SMC), Republic of Korea. Eligible participants were individuals with T1D enrolled in the national home care pilot program and using CGM. Between July and August 2024, 18 volunteers were recruited and randomized in a 1:2 ratio to the control and intervention groups. Over 6 weeks, participants in the intervention group received weekly personalized coaching messages based on CGM data for glycemic management and biweekly emotional support messages tailored to depressive symptoms assessed using the Patient Health Questionnaire (PHQ-9). In contrast, the control group continued usual care under the national home care program, including at least bi-monthly remote educational consultations. All participants provided written informed consent before participation. The study adhered to the Declaration of Helsinki and Korean Good Clinical Practice, with approval from the SMC Institutional Review Board (2024-06-077). The study was registered at ClinicalTrials.gov (NCT06525454).

### Study participants and randomization

Eligible participants were adults aged 20–69 years with T1D, already enrolled in the National Home Care Pilot program, through which they received in-person education and bi-monthly remote consultations via phone or interactive messaging. All participants underwent CGM as recommended by their healthcare providers, were on multiple insulin injections, had access to smartphones, and volunteered to participate. Individuals taking oral glucose-lowering medications, steroids, or immunosuppressants, those undergoing anticancer treatment, or those who were pregnant or breastfeeding were excluded. A pre-specified random allocation sequence was generated through R, and participants were block-randomized 3 × 6 in a 1:2 ratio to the control and intervention groups. A 1:2 allocation ratio was selected to reflect the feasibility-oriented nature of this pilot trial. In the absence of a formal sample size calculation, the target sample size of 18 participants was determined based on clinical and practical considerations. A higher proportion of participants were allocated to the intervention group to allow more comprehensive assessment of intervention delivery, adherence, and patient engagement.

### Intervention

Patients in the intervention group were invited to a 1:1 chat room on a social media platform, where they received semiautomated coaching messages generated by a program developed by the research team. These messages replaced the standard remote educational consultations, typically conducted bi-monthly via phone calls or interactive messaging, under the national home care pilot program. The messages offered individualized guidance on glycemic control based on each patient’s CGM data. The program consisted of two components: glycemic management (CGM part) and depressive symptom management (depression part), with biweekly PHQ-9 surveys to assess depressive symptoms. The CGM component included summaries of CGM data, insulin adjustment suggestions, and recommendations for dietary modifications and physical activity. Weekly summaries of key CGM metrics, along with guidance on insulin titration, dietary recommendations, and physical activity suggestions. Weekly summary of CGM key metrics and daily CGM trend graphs for one week, were generated, with bar plots visualizing changes in CGM metrics compared to the previous week. The CGM feedback algorithm was structured as a two-step, rule-based decision-making process. In the metrics part, CGM data were evaluated to quantify the proportion of time spent within, above, or below the target glucose range. In the insulin part, the algorithm considered the rate, duration, and timing of glucose excursions to determine appropriate insulin adjustments and deliver personalized coaching messages, with the overall algorithm structure and detailed decision rules summarized in Figure S3 and Table S2. Messages were tailored to indicate whether CGM targets were met and provided feedback on unmet goals. Guidance on dietary modifications and physical activity, along with insulin dose adjustment recommendations, were based on CGM patterns, covering both long- and rapid-acting insulin. The messages, sent weekly with varied phrasing to maintain a personalized tone, also included encouraging content to promote patient motivation. In the depressive symptom monitoring, depressive symptoms were classified as normal, mild, moderate, or severe, and further categorized into four subdomains: affective, somatic, internalizing, and sensorimotor [13]. A biweekly PHQ-9 survey was used to track changes in these subdomains, with semiautomated messages generated. Visual feedback was provided, indicating the participant’s current depressive severity and comparing subdomain scores with previous assessments. All algorithms used were rule-based, developed based on clinical expertise and literature review.

### Data collection

Demographic, anthropometric, and laboratory data were collected from all participants. During the first visit, data regarding demographic characteristics, smoking status, alcohol consumption, and year of CGM initiation were obtained using self-administered questionnaires. Smoking status was categorized as never smoked, former smoker, or current smoker. Alcohol consumption was categorized as none or current drinker. HbA1c levels were measured at the first visit. The estimated glomerular filtration rate (eGFR) was determined using the Chronic Kidney Disease Epidemiology Collaboration 2021 (CKD-EPI 2021). CGM data were collected from all participants over a 6-week period. Patient-reported outcomes included the PHQ-9 and the Diabetes Treatment Satisfaction Questionnaire status version (DTSQs), which were collected at baseline and at the end of the study period, while the Diabetes Treatment Satisfaction Questionnaire change version (DTSQc) was administered at follow-up. For participants in the intervention group, additional PHQ-9 assessments were conducted biweekly to monitor depressive symptoms and provide tailored messaging content. In addition, the number of remote messages sent by diabetes care professionals for educational consultations, as well as those initiated by participants for management-related inquiries, was evaluated over the 6-week period.

### Outcomes

Feasibility was evaluated by comparing the time in range (TIR; 70–180 mg/dL) at 6 weeks between the intervention and control groups receiving standard care under the national home care pilot program. Other CGM-derived metrics (time above range [TAR] > 250 mg/dL, TAR > 180 mg/dL, time below range [TBR] < 70 mg/dL, TBR < 54 mg/dL, coefficient of variation [CV], glucose management indicator [GMI], time in tight range [TITR]), sensor wear time at week six, and patient-reported outcomes (PHQ-9 and DTSQ scores) were evaluated. Additionally, the number of remote messages sent by diabetes care professionals and participants was evaluated over the 6-week period. In addition to CGM-derived outcomes, feasibility was assessed based on recruitment, retention, intervention adherence, CGM sensor wear time, and patient engagement during the study period.

### Statistical analysis

Sample size calculation based on effect size was not considered because this study aimed to identify the acceptability and feasibility of the intervention. Therefore, a minimum sample size of 18 individuals (6 in the control group and 12 in the intervention group) was recruited. Continuous variables were compared between the intervention and control groups using the Mann–Whitney test due to the small sample size. Categorical variables were analyzed using the chi-square test to report baseline differences between groups. A Mann–Whitney test was used to compare the median TIR (70–180 mg/dL) at week 6 and other CGM metrics, including CV, GMI, sensor wear time, TITR (70–140 mg/dL), TAR (> 180 mg/dL), TAR (> 250 mg/dL), TBR (< 70 mg/dL), TBR (< 54 mg/dL), and PHQ-9 and DTSQ scores at week 6, as well as the number of remote messages over the 6 weeks between the intervention and control groups. Additionally, Wilcoxon signed-rank tests were used to assess within-group changes from baseline to week 6. Effect sizes (Cohen’s *d*) and corresponding 95% confidence intervals were calculated for between-group comparisons. All statistical analyses were performed using R software, version 4.3.3 (R Foundation for Statistical Computing). Statistical significance was set at *p* < 0.05.

## Results

### Baseline characteristics

**Fig. 1:**
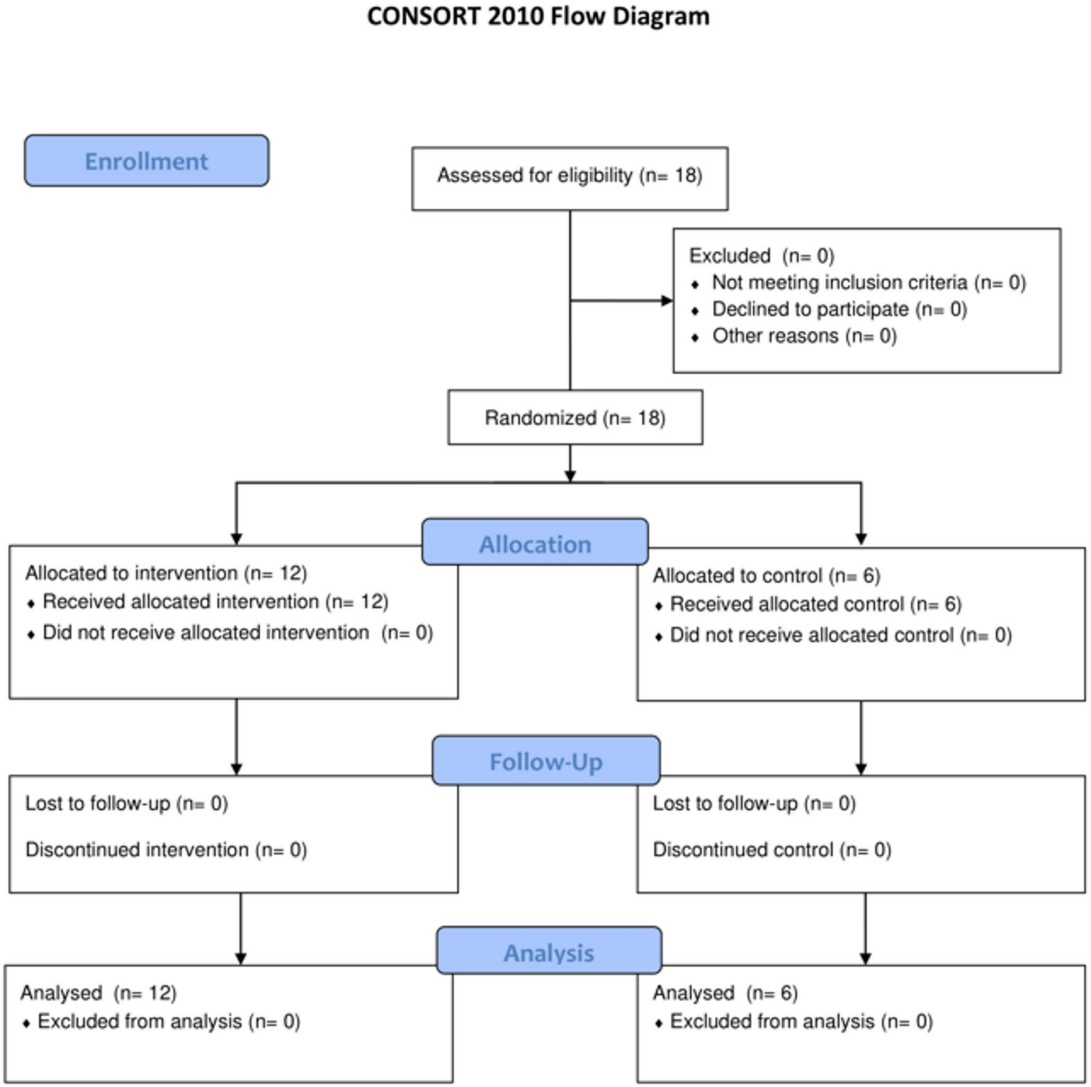
Flow diagram

Of the 18 participants, 6 and 12 were randomly assigned to the control and intervention groups, respectively (Fig. 1). No significant differences in covariates were observed between the intervention and control groups (*p* > 0.05, Table 1). The median age was 34 years (IQR: 26–50), 72% of participants were female, and 6% were identified as current smokers and drinkers. The median baseline HbA1c was 6.4% (IQR: 6.0%–7.2%), and the eGFR was 105.8 mL/min/1.73 m² (IQR: 82.5–115.3 mL/min/1.73 m²). Baseline CGM sensor wear time was 92.5% (IQR: 70.0%–97.6%), TIR was 79.2% (IQR: 74.7%–82.1%), and CV was 31.1% (IQR: 28.7%–35.1%), indicating well-controlled T1D.

**Table 1:** Baseline characteristics of study participants

	Control (*N* = 6)	Intervention (*N* = 12)	*P*-value
Age, years	30.0 [23.0; 40.0]	34.0 [27.5; 54.5]	0.542
*Sex*			1.000
Male	2 (33.3%)	3 (25.0%)	
Female	4 (66.7%)	9 (75.0%)	
*Smoking status*			1.000
Never	6 (100.0%)	11 (91.7%)	
Ever	0 (0.0%)	1 (8.3%)	
Current	0 (0.0%)	0 (0.0%)	
*Alcohol consumption*			1.000
Non-drinker	6 (100.0%)	11 (91.7%)	
Current drinker	0 (0.0%)	1 (8.3%)	
Time since T1D diagnosis, years	11.5 [9.0; 13.0]	6.5 [3.0; 14.0]	0.348
*CGM device type*,* n (%)*			0.079
Caresense	1 (16.7%)	1 (8.3%)	
Dexcom	1 (16.7%)	8 (66.7%)	
Diaconn	1 (16.7%)	0 (0.0%)	
Libre	3 (50.0%)	1 (8.3%)	
Medtronic	0 (0.0%)	2 (16.7%)	
*Year of CGM initiation*,* n (%)*			0.061
2017	1 (16.7%)	0 (0.0%)	
2019	3 (50.0%)	6 (50.0%)	
2020	2 (33.3%)	0 (0.0%)	
2023	0 (0.0%)	5 (41.7%)	
2024	0 (0.0%)	1 (8.3%)	
Sensor wear time (%)	88.5 [81.0; 97.2]	92.5 [70.0; 98.2]	0.892
TIR (70–180 mg/dL)	76.2 [61.0; 78.2]	81.0 [77.1; 85.4]	0.067
TAR (> 180 mg/dL)	21.2 [15.5; 32.7]	17.4 [8.6; 21.5]	0.250
TBR (< 70 mg/dL)	2.9 [0.0; 6.3]	1.2 [0.3; 4.2]	0.814
HbA1c, %	6.6 [6.6; 7.2]	6.2 [5.8; 6.9]	0.174
eGFR, mL/min/1.73 m^2^	105.3 [93.7; 118.4]	108.8 [75.9; 115.0]	0.808

Values are presented as medians [interquartile ranges] for continuous variables and n (%) for categorical variables

CGM, continuous glucose monitoring; HbA1c, glycosylated hemoglobin; eGFR, estimated glomerular filtration rate

### CGM metric outcomes

**Fig. 2:**
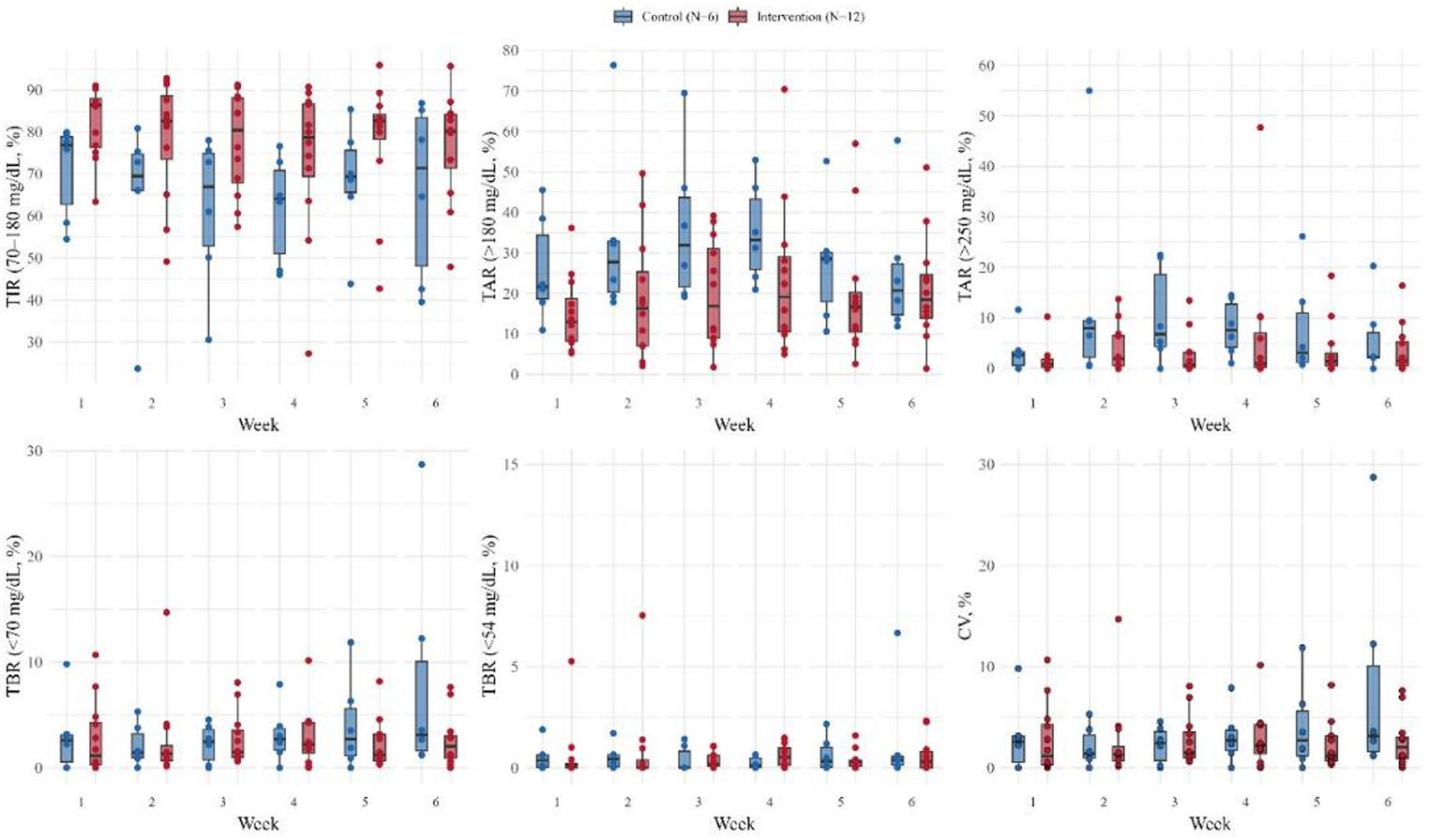
Trends in continuous glucose monitoring metrics, including time in range (TIR), time above range (TAR), time below range (TBR), and coefficient of variation (CV), over 6 weeks in patients with type 1 diabetes randomized to the control or intervention group

At baseline, median TIR (70–180 mg/dL) was 76.2% (IQR: 61.0–78.2) in the control group and 81.0% (IQR: 77.1–85.4) in the intervention group (*p* = 0.067). At week 6, median TIR was 72.6% (IQR: 46.8–77.0) in the control group and 79.0% (IQR: 65.0–82.1) in the intervention group, with a median difference of 7.5% between the groups, which was not statistically significant (*p* = 0.083) (Table 2). A moderate effect size was observed for TIR at week 6 (Cohen’s *d* = 0.55, 95% CI − 0.46 to 1.54), indicating a possible improvement in glycemic control with substantial uncertainty due to wide confidence intervals. For other CGM metrics, median TAR (> 180 mg/dL) was 21.8% in the control group and 18.1% in the intervention group (median difference: 6.1%), while median TBR (< 70 mg/dL) was 3.0% and 1.9%, respectively (median difference: 0.8%), with no significant between-group differences (Table S1). Over the 6-week period, CGM metrics, including TIR, TAR, TBR, and CV, remained stable in both groups, with no significant differences observed (Fig. 2). These findings, demonstrating comparable outcomes between the intervention and control groups receiving the current treatment, support the feasibility of a semiautomated intervention as an alternative to care under the national home care pilot program.

**Table 2:** Primary outcomes

	Control (*N* = 6)	Intervention (*N* = 12)	Median difference (95% CI)	*P*-value	Effect size (95% CI)
TIR (70–180 mg/dL) at baseline, %	76.2 [61.0; 78.2]	81.0 [77.1; 85.4]	−6.16 (− 19.75 to 0.23)	0.067	
TIR (70–180 mg/dL) at week 6, %	72.6 [46.8; 77.0]	79.0 [65.0; 82.1]	−7.74 (− 31.3 to 6.01)	0.083	0.55 (−0.46 to 1.54)

Values are presented as medians [interquartile ranges]

TIR, time in range

### Patient-reported outcomes

Table 3 presents results of depressive symptom assessments using the PHQ-9, treatment satisfaction measured by the DTSQ, and the number of remote messages sent by diabetes care professionals for educational consultations and by participants for management-related inquiries over a 6-week period. At baseline, median PHQ-9 scores were 7.0 (IQR: 4.0–8.0) in the control group and 6.0 (IQR: 3.0–8.5) in the intervention group (*p* = 0.740). After 6 weeks, the intervention group demonstrated a significant reduction in PHQ-9 scores (2.5, IQR: 1.5–4.5) compared to the control group (6.0, IQR: 4.0–9.0), with a between-group difference of − 3.0 (CI: 1.0–7.0), indicating improved depressive symptoms (Table 3). A large effect size for PHQ-9 sum score at week 6 (Cohen’s d = − 1.30, 95% CI − 2.36 to − 0.21) was observed, suggesting a decrease in depressive symptoms with the intervention. In the pre-post analysis, PHQ-9 scores significantly decreased to 3.0 (95% CI: 7.5–1.5) in the intervention group (*p* < 0.001), with no significant change observed in the control group (*p* = 1.000). No significant difference in endpoint DTSQs was observed between the two groups, although the intervention group showed increased satisfaction with a median DTSQc of 2.0 (IQR: 1.7–2.2). Over the 6-week period, the total number of remote messages was significantly higher in the intervention group (34.0, IQR: 30.5–40.5) than in the control group (2.0, IQR: 2.0–3.0; *p* < 0.001). Diabetes care professional-generated messages accounted for a median of 30.0 (IQR: 28.5–33.0) in the intervention group versus 2.0 (IQR: 2.0–3.0) in the control group. Patient-generated messages were also significantly higher in the intervention group (3.5, IQR: 1.0–5.5) compared to 0.0 (IQR: 0.0–0.0) in the control group. These findings suggest that the semiautomated program may facilitate more frequent remote communication.

**Table 3:** Results of patient-reported outcomes

	Control (*N* = 6)	Intervention (*N* = 12)	Median difference (95% CI)	*P*-value	Effect size (95% CI)
PHQ-9 sum score at baseline	7.0 [4.0; 8.0]	6.0 [3.0; 8.5]	0 (− 2.0 to 4.0)	0.740	
PHQ-9 sum score at week 6	6.0 [4.0; 9.0]	2.5 [1.5; 4.5]	3 (1.0 to 7.0)	0.030	− 1.30 (− 2.36 to − 0.21)
DTSQs sum score at baseline	35.0 [28.0; 39.0]	35.0 [27.5; 39.5]	0 (− 9.0 to 7.0)	1.000	
DTSQs sum score at week 6	34.5 [28.0; 39.0]	36.0 [33.5; 42.0]	−3 (− 9.0 to 5.0)	0.450	0.30 (− 0.69 to 1.29)
Message count Total	2.0 [2.0; 3.0]	34.0 [30.5; 40.5]		< 0.001	
Patient-generated	0.0 [0.0; 0.0]	3.5 [1.0; 5.5]		< 0.001	
Healthcare provider-generated	2.0 [2.0; 3.0]	30.0 [28.5; 33.0]		< 0.001	

Values are presented as medians [interquartile ranges]

CGM, continuous glucose monitoring; TIR, time in range; TAR, time above range; TBR, time below range; PHQ-9, Patient Health Questionnaire-9; DTSQs, Diabetes Treatment Satisfaction Questionnaire

### Feasibility and adherence outcomes

All eligible individuals agreed to participate, and no participants withdrew or were lost to follow-up during the 6-week study period. Sensor wear time of CGM remained high in both groups throughout the study. Participants in the intervention group demonstrated high engagement, as evidenced by a substantially higher frequency of bidirectional messaging compared with the control group (Table 3).

## Discussion

In this 6-week randomized controlled feasibility trial, individualized automated coaching interventions for glycemic and emotional support were found to be both acceptable and feasible in individuals with well-controlled T1D. Glycemic outcomes, including TIR, TAR, and TBR at week 6, were comparable between the intervention and control groups, indicating similar levels of glucose control. Although the intervention group showed slightly higher TIR and lower TAR and TBR, these differences were not statistically significant. The PHQ-9 scores in the intervention group showed a median decrease of three points, indicating a meaningful improvement in depressive symptoms. Despite similar endpoint DTSQs scores between groups, the intervention group exhibited significantly greater patient engagement, with higher messaging activity and a marked improvement in treatment satisfaction as assessed by the DTSQc. These findings support the potential of semiautomated interventions to promote more frequent remote interactions and reduce the need for intensive CGM monitoring by healthcare professionals, offering a viable alternative to the current national homecare pilot program for T1D. By automating data interpretation and feedback delivery through an algorithm, the system alleviated the burden on healthcare professionals and enable more efficient allocation of human resources, allowing clinical teams to focus on areas requiring direct, personalized care [7]. Digital therapeutics are evidence-based, software-driven interventions developed to prevent, manage, or treat medical conditions and often undergo formal regulatory evaluation, as exemplified by diabetes-focused platforms such as Welldoc^®^ or mental health programs like HelloBetter^®^ Diabetes [14, 15]. While the present CGM-based coaching system shares core principles of digital therapeutics—namely data-driven personalization and behavior support—it was designed as a semiautomated adjunct to existing care pathways rather than a standalone, regulatory-cleared digital therapeutic.

Previous studies have shown that remote monitoring using CGM data, along with coaching and support programs delivered via telemedicine, can improve glucose management [16]. Although the previous study was a case study, the present study was a randomized controlled trial (RCT) designed to assess the feasibility of personalized coaching messages. The treatment group had a higher TIR than the control group, although the difference was not statistically significant due to the small sample size. In a prior RCT, patients with type 2 diabetes using basal insulin who received a rule-based algorithm for insulin titration through voice-based artificial intelligence demonstrated significantly better insulin adherence and reduced diabetes distress compared to a standard-of-care control group [17]. The primary distinction from previous studies is that the present study delivered coaching messages for basal and premeal insulin titration based on actual glucose fluctuations derived from a week of CGM data. Another study compared treatment guided by SMBG with that guided by CGM in patients requiring insulin and reported fewer hypoglycemic events in the CGM group [18]. In patients requiring premeal insulin injections, glycemic variability can be substantial, and previous research found only a moderate correlation between the SD of SMBG and the SD of CGM (*r* = 0.54, *p* < 0.05) [19]. Therefore, in patients requiring rapid-acting insulin, titration coaching may be more accurate when based on CGM rather than SMBG data. In the present study, patients received personalized coaching messages and had the option of engaging in one-on-one discussions with a nurse if they had questions. Remote monitoring, feedback, and interaction with healthcare professionals can contribute to improved glycemic control [20]. In previous studies, coaching for families of children with T1D and personalized programs for patients to reduce diabetes distress did not result in a significant reduction in emotional burden in the treatment group compared to the control group but did lead to reduced distress and improved self-management behaviors in the treatment group [21, 22]. A scoping review evaluating the impact of health coaching programs on patients with type 2 diabetes reported significant effects on glucose management and self-care behaviors but found no association with improvements in psychosocial outcomes [23]. However, a study assessing the effectiveness of a mobile app designed to enhance emotional well-being and self-management in people with type 2 diabetes, who were medical beneficiaries and had mild-to-moderate depressive symptoms, reported a significant reduction in the severity of depression and perceived stress [24]. In our study, depressive symptoms improved markedly in the intervention group relative to the control group; however, the long-term effectiveness of this approach remains to be confirmed in larger, longer-duration RCTs.

### Strengths and limitations

We evaluated the feasibility and acceptability of a novel semiautomated delivery program for insulin titration coaching messages for basal and prandial insulin, as well as personalized monitoring feedback and summary plots based on weekly CGM data in adults with T1D, in a randomized trial. Depressive symptoms were also monitored biweekly using a validated questionnaire, and coaching messages were tailored to specific subdomains of depression identified through this process. To our knowledge, this is the first RCT to demonstrate the feasibility and acceptability of a semiautomated system that preprocesses and analyzes repeatedly measured CGM and psychological data and delivers integrated, personalized coaching messages targeting both glycemic control and emotional well-being in adults with T1D. The structured nature of this program, including regular remote consultations by a multidisciplinary team, represents a strong active comparator and may have limited the ability to detect incremental improvements in glycemic outcomes attributable to the semiautomated intervention. However, this study has some limitations. As this was a feasibility trial, the overall sample size was relatively small, which may limit the generalizability of the findings. In addition, the follow-up duration was only 6 weeks. Larger RCTs with more participants and extended follow-up periods are warranted to validate the effectiveness and long-term impact of this intervention. Given the small sample size, short time of follow-up and feasibility-oriented design, this study was not powered to detect differences in glycemic outcomes, and any observed trends should be interpreted descriptively rather than as evidence of effectiveness. However, the absence of deterioration in glycemic metrics, together with high retention and engagement, supports the feasibility of implementing this semiautomated intervention as an alternative care model. The unequal randomization ratio, while enabling richer evaluation of feasibility and acceptability in the intervention group, may have introduced group imbalance and limited the precision of between-group comparisons, warranting descriptive interpretation of glycemic outcomes and confirmation in larger trials with balanced allocation. Finally, the study population consisted predominantly of adults with relatively well-controlled T1D, as reflected by low baseline HbA1c levels and high TIR. This likely introduced a ceiling effect, limiting the potential to observe further improvement in glycemic outcomes. Consequently, the generalisability of these findings to individuals with suboptimal glycemic control or greater glycemic variability may be limited, and future studies should evaluate this intervention in more diverse T1D populations.

## Conclusions

This study demonstrates the feasibility and acceptability of a semiautomated, CGM-based coaching intervention that supports glycemic and emotional management in adults with T1D without compromising glycemic control. By leveraging rule-based algorithms and routinely collected CGM data, this approach offers a scalable and potentially sustainable model that can complement existing diabetes care systems while reducing reliance on intensive human resources. Such a framework may facilitate broader implementation across healthcare settings with established CGM use. Further studies will be needed to determine how this approach could be evaluated, adapted, and implemented within different regulatory and healthcare contexts, including settings outside Korea. Individualized automated coaching messages for glycemic and emotional management were acceptable and feasible for individuals with T1D. Comprehensive monitoring and coaching of glycemic control and emotional well-being may contribute to improved emotional management.

## Supplementary Information

Below is the link to the electronic supplementary material.

Supplementary Material 1

## Data Availability

Data supporting the conclusions of this study are included in the article and its additional files.
