# Supplementary material for: Feasibility of a semiautomated, individualized coaching intervention for glycemic management in adults with type 1 diabetes: a pilot randomized controlled trial
Source: J Endocrinol Invest. 2026 Mar 7;49(7):1683–92. doi: 10.1007/s40618-026-02835-1 (PMC13451404; doi:10.1007/s40618-026-02835-1)
Supplement: Supplementary file 1 [file 40618_2026_2835_MOESM1_ESM.docx]

**Table S1. CGM metrics**

|  | Control (N = 6) | Intervention (N = 12) | Median Difference (95% CI) | P-value | Effect size (95% CI) |
| --- | --- | --- | --- | --- | --- |
| Sensor wear time at week 6, % | 97.1 [90.0; 99.3] | 96.2 [89.8; 97.4] | 0.84 (−7.51 to 6.92) | 0.542 | 0.42 (-0.58 to 1.40) |
| CV at week 6, % | 34.8 [32.6; 40.4] | 32.3 [27.2; 35.2] | 3.74 (−1.6 to 8.7) | 0.174 | -0.87 (-1.88 to 0.17) |
| GMI at week 6, % | 6.8 [6.6; 7.4] | 6.8 [6.6; 7.0] | 0.12 (−0.33 to 0.78) | 0.743 | -0.28 (-1.26 to 0.71) |
| TITR (70–140 mg/dL) at week 6, % | 45.6 [25.7; 51.1] | 51.0 [39.0; 57.9] | −6.99 (−27.14 to 7.59) | 0.242 | 0.50 (-0.50 to 1.49) |
| TAR (>180 mg/dL) at week 6, % | 21.8 [21.3; 44.5] | 18.1 [13.9; 30.2] | 6.13 (−6.8 to 27.38) | 0.242 | -0.46 (-1.45 to 0.54) |
| TAR (>250 mg/dL) at week 6, % | 3.1 [2.7; 14.1] | 1.9 [1.0; 4.6] | 1.6 (−1.22 to 12) | 0.122 | -0.28 (-1.26 to 0.71) |
| TBR (<70 mg/dL) at week 6, % | 3.0 [1.3; 8.8] | 1.9 [1.0; 3.2] | 0.75 (−1.88 to 6.98) | 0.325 | -0.37 (-1.35 to 0.63) |
| TBR (<54 mg/dL) at week 6, % | 0.5 [0.4; 0.8] | 0.5 [0.3; 0.7] | 0.01 (−0.44 to 0.5) | 0.963 | 0.12 (-0.86 to 1.10) |

Values are presented as medians [interquartile ranges]. Abbreviations: CGM, continuous glucose monitoring; CV, coefficient of variance; GMI, glucose management indicator; TITR, time in tight range; TAR, time above range; TBR, time below range

**Table S2. CGM message case classification**

| Metrics part | |
| --- | --- |
| Case1 | All glycemic range goals (TIR, TAR, TBR) satisfied |
| Case2 | Sensor wear goal satisfied (≥70%) |
| Case3 | Only TIR goal satisfied |
| Case4 | TIR goal not satisfied |
| Insulin part | |
| Case1 | Glucose <70 mg/dL sustained for ≥15 min on at least two nights during 00:00–06:00. |
| Case2 | For ≥3 consecutive days, TAR (>180 mg/dL) ≥25% during 00:00–06:00, nocturnal minimum ≥100 mg/dL, and 06:00–08:00 glucose ≥150 mg/dL, with ≤1 meal during 23:00–06:00. |
| Case3 | TBR (<70 mg/dL) exceeding 4% of total time on at least three days (00:00–24:00) |
| Case4 | TAR (>250 mg/dL) exceeding 10% of total time on at least four days during 10:00–24:00. |
| Case5 | At least four postprandial episodes (10:00–24:00) with glucose peaks >220 mg/dL following meals. |
| Case5-1 | During 08:00–24:00, postprandial glucose returns to 70–140 mg/dL within 5 h after a meal. |
| Case5-2 | During 08:00–24:00, postprandial glucose remains >140 mg/dL within 5 h after a meal. |
| Case6 | During 00:00–03:00, at least two nights with a ≥100 mg/dL glucose drop within 4 h after TAR (>250 mg/dL). |
| EhyperE | On at least three days per week, TAR (>250 mg/dL) sustained for ≥2 h (00:00–24:00). |

**Figure S1. Effect sizes (Cohen’s d) and 95% confidence intervals for between-group differences in key outcomes.**

**Figure S2. Message templates**

**Figure S3. Decision making process**
